# Differential Response of Primary Alveolar Type I and Type II Cells to LPS Stimulation

**DOI:** 10.1371/journal.pone.0055545

**Published:** 2013-01-31

**Authors:** Mandi H. Wong, Meshell D. Johnson

**Affiliations:** 1 San Francisco Veterans Affairs Medical Center, San Francisco, California, United States of America; 2 Northern California Institute for Research and Education, San Francisco, California, United States of America; 3 Department of Medicine, University of California San Francisco, San Francisco, California, United States of America; Louisiana State University, United States of America

## Abstract

The alveolar epithelium serves as a barrier between organism and environment and functions as the first line of protection against potential respiratory pathogens. Alveolar type II (TII) cells have traditionally been considered the immune cells of the alveolar epithelium, as they possess immunomodulatory functions; however, the precise role of alveolar type I (TI) cells, which comprise ∼95% of the alveolar epithelial surface area, in lung immunity is not clear. We sought to determine if there was a difference in the response of TI and TII cells to lung injury and if TI cells could actively participate in the alveolar immune response. TI cells isolated via fluorescence activated cell sorting (FACS) from LPS-injured rats demonstrated greater fold-induction of multiple inflammatory mediators than TII cells isolated in the same manner from the same animals. Levels of the cytokines TNF-α, IL-6 and IL-1β from cultured primary rat TI cells after LPS stimulation were significantly increased compared to similarly studied primary rat TII cells. We found that contrary to published reports, cultured TII cells produce relatively small amounts of TNF-α, IL-6 and IL-1β after LPS treatment; the higher levels of cytokine expression from cultured TII cells reported in the literature were likely from macrophage contamination due to traditional non-FACS TII cell isolation methods. Co-culture of TII cells with macrophages prior to LPS stimulation increased TNF-α and IL-6 production to levels reported by other investigators for TII cells, however, co-culture of TI cells and macrophages prior to LPS treatment resulted in marked increases in TNF-α and IL-6 production. Finally, exogenous surfactant blunted the IL-6 response to LPS in cultured TI cells. Taken together, these findings advocate a role for TI cells in the innate immune response and suggest that both TI and TII cells are active players in host defense mechanisms in the lung.

## Introduction

The alveolar epithelium is an important component of the innate immune response of the lung. By providing an anatomic barrier that separates the organism from the external environment, the alveolar epithelium serves as a first line of defense against potential inhaled pathogens. While the cells of the innate immune system, such as alveolar macrophages and dendritic cells, harbor the bulk of the responsibility for prompting an immune reaction upon encountering inhaled pathogens, the cells that comprise the alveolar epithelium have also been implicated in aiding to trigger an inflammatory response.

The alveolar epithelium is comprised of two morphologically different cell types – alveolar type I (TI) and alveolar type II (TII) cells. TII cells, which cover 3–5% of the alveolar surface area, are cuboidal epithelial cells with diameters of ∼10 µm. TII cells have been extensively studied and have been labeled defenders of the alveolus for their immunomodulatory functions [1]. TII cells can produce cytokines and chemokines, such as TNF-α, IL-6, IL-1β, monocyte chemoattractant protein 1 (MCP-1), macrophage inflammatory protein 1α (MIP-1α), growth related oncogene α (GRO-α) and granulocyte-macrophage colony stimulating factor (GM-CSF) in response to various forms of lung injury induced by bacteria, viruses, or mechanical ventilation [2]–[4]. TII cells also produce, secrete, and recycle surfactant, which can enhance chemotaxis, bacterial uptake and phagocytosis by alveolar macrophages [5], [6], but can also inhibit cytokine production in response to LPS [7]. Far less is known about the potential of TI cells to participate in the inflammatory response. TI cells are large, thin squamous epithelial cells with diameters that range up to 100 µm and cell bodies that can be as thin as 50 nm [8]. TI cells comprise 95% of the alveolar epithelium, making them a major component of the physical barrier to respiratory pathogens. Despite this fact, the prevailing paradigm has been that TII cells are the inflammatory cells of the alveolar epithelium, while TI cells help form the mechanical barrier to pathogens but do not participate in the active cellular immune response of the lung.

Newer data led us to reconsider the current thoughts surrounding the role of TI cells in alveolar inflammation. TI cells contain toll-like receptor 4 (TLR4), a receptor for lipopolysaccharide (LPS), a cell wall protein found on gram negative bacteria, and primary rat TI cells have been shown to produce the pro-inflammatory cytokines TNF-α, IL-6 and IL-1β in response to LPS stimulation [9]. Given these findings, particularly in the context of the extensive alveolar surface area covered by TI cells, we hypothesized that TI cells were capable of producing a wide array of inflammatory mediators like their TII counterparts and that the alveolar microenvironment can influence TI cell cytokine production.

We used fluorescence-activated cell sorting (FACS) to isolate relatively pure populations of TI and TII cells for our studies. We performed PCR array analysis on primary rat TI and TII cells isolated by FACS from LPS-injured and non-injured control animals to compare their inflammatory profiles. To isolate the specific responses to LPS treatment from each cell type, we cultured FACS-isolated TI and TII cells, treated the cells with LPS, and then measured pro-inflammatory cytokine secretion using ELISA. We discovered that TI cells produce more cytokines per cell after LPS treatment than TII cells, but this effect was magnified by the fact that TII cells produced relatively small amounts of TNF-α, IL-6 or IL-1β in response to LPS, which is contrary to previously published reports [10]–[12]. We discovered that the discrepancies between our results and those of other investigators were due to the method of TII cell isolation, and that contamination of TII cell preparations by macrophages accounted for the observed differences in cytokine expression. Alveolar macrophages are dominant mediators of lung immunity, patrolling the epithelial surface to phagocytize invading pathogens and generating cytokines and chemokines to recruit neutrophils to augment the immune response [13]. Given the differences seen in the response of TI and TII cells to LPS stimulation and the effect of co-culturing TII cells with macrophages, we sought to evaluate how elements of the alveolar microenvironment could affect TI cells. Therefore, we also examined the effects of macrophages and surfactant on the LPS-induced inflammatory response of TI cells.

The studies contained in this report not only suggest that TI cells are capable of mounting an inflammatory response after LPS exposure, but that the microenvironment surrounding both TI and TII cells may contribute to their ability to secrete cytokines and participate in the innate immune response. Determining how TI cells respond to respiratory pathogens and clarifying their role in the immune response in the lung may alter how lung immunity is viewed and possibly produce new therapeutic targets for addressing inflammation in the distal lung.

## Methods

### Ethics statement

All animal experiments were performed in accordance with, and with the approval of, protocols sanctioned by the IACUC committees at the University of California, San Francisco and the San Francisco VA Medical Center (approval #10-075-01).

### Animal studies

Pathogen-free, adult male Sprague-Dawley rats from Charles River Laboratories (Hollister, CA) weighing ∼150–200 g were used in all experiments. All animals were housed and provided *ad libitum* access to food and water. Rats were anesthetized by brief inhalation of 4% isoflurane prior to intratracheal instillation of normal saline (control) or LPS 10 mg/kg (Escherichia coli endotoxin serotype O127:B8 (Sigma-Aldrich, St. Louis, MO)) [14] through a 24-gauge angiocatheter inserted into the trachea. After instillation, the animals were allowed to recover from the anesthesia, and were euthanized at various timepoints with an intraperitoneal injection of pentobarbital (100 mg/kg) before removal of the lungs.

### Cell isolation

#### FACS isolation of alveolar TI and TII cells

Adult Sprague Dawley rats were euthanized with an intraperitoneal injection of pentobarbital (100 mg/kg). Alveolar TI and TII cells were isolated from the lungs by FACS using methods described by Gonzalez et al [15]. After enzymatic digestion with porcine grade IV elastase (Sigma), the lungs were minced to produce a single cell suspension that was then subjected to FACS using alveolar epithelial cell antibodies that are specific, within the lung, to the apical membranes rat TI (RTI40) [16] or rat TII (RTII70) [17] cells (kind gifts from Dr. Leland Dobbs, UCSF). One rat was used for each isolation; the typical yield was ∼1.5×10^6^ TI cells or 8×10^6^ TII cells per animal. Cell purity was determined via immunocytochemical staining of samples using RTI40 to label TI cells and RTII70 to label TII cells. Cell preparations containing >2% of contaminating cells were discarded; >95% of all cells were viable as measured by a Live/Dead kit (Invitrogen, Carlsbad, CA).

#### Non-FACS isolation of alveolar TII cells

TII cells were isolated using a conventional method of enzymatic digestion with porcine elastase followed by panning over plates coated with IgG to remove macrophages and leukocytes, as described previously [17]. Preparations containing <85% TII cells or >2% TI cells were discarded. Cell purity was determined by cell-specific staining with the appropriate antibodies to TII cells and DAPI, and >98% of all cells were viable as measured by a Live/Dead kit (Invitrogen).

#### Macrophages

The lungs of adult Sprague-Dawley rats were lavaged 5 times with 7 ml aliquots of Ca^+2^/Mg^+2^-free PBS containing 5 mM EDTA and 5 mM EGTA at 37°C. The aliquots for each rat were pooled and centrifuged at 500 g for 12 minutes. The cells were then collected, counted, and resuspended in DMEM containing 1% penicillin-streptomycin and 10% fetal bovine serum (Hyclone, Waltham, MA). In order to assess purity of the preparations, cells were stained with a modified Wright Giemsa stain, and reached purities of >95% macrophages.

### Cell culture

Using the methods of Gonzales et al [15] and Wang et al [18], TI cells isolated via FACS and were resuspended in DMEM-H16 containing 20% fetal bovine serum. The cells were then cultured on Transwell plates at a density of 500,000 cells/well and allowed to recover from the isolation and FACS procedures overnight in a 10%CO_2_/air incubator at 37°C. TII cells were resuspended in DMEM-H16 with 10% fetal bovine serum, plated at the same density, and cultured in 5% CO_2_ at 37°C [19]. The next day, LPS 10 ug/ml (Sigma) was added to the cells in serum-free media. Cells were cultured for 18 hours prior to collection of supernatant and cells. For studies that were focused on the NF-κB/IκB pathway, cells were isolated from the lung in the exact same manner as above, except they were not subject to FACS. Instead, the cells were cultured on glass slides overnight before treatment with LPS 10 ug/ml for varying lengths of time – 10 minutes, 30 minutes, 1 hour, 4 hours, 8 hours and 18 hours. Cells were then spun at 700 rpm to adhere to the slide before fixation with paraformaldehyde and subsequent staining with antibodies as described below.

Macrophages collected from the bronchoalveolar lavage were added to cultured TI or TII cells (cultured at a density of 500,000 cells/well) in increasing numbers to represent cultures containing 5% (25,000), 10% (50,000), 15% (75,000) or 20% (100,000) macrophages. Similar numbers of macrophages were cultured alone as a control. For co-cultures of alveolar epithelial cells and macrophages, 10% macrophages, equaling 50,000 macrophages, were added.

### RT^2^ Profiler PCR Array and Quantitative PCR

Approximately 2 µg total RNA were mixed with genomic DNA elimination buffer prior to synthesis of cDNA using the RT^2^ First Strand Kit (SABiosciences, Valencia, CA). The resulting cDNA reaction was mixed with SYBR green qPCR master mix and transferred into the 96-well RT^2^ Profiler PCR Array for Rat Inflammatory Cytokines and Receptors (SABiosciences), containing appropriate primers for genes associated with the chemokines, cytokines, and interleukins involved in the inflammatory response as well as their receptors and primers for housekeeping genes. Real-time PCR utilizing the RT^2^ Profiler PCR Array System was performed according to manufacturer's instructions utilizing an ABI Prism 7500 RT-PCR thermocycler (Applied Biosystems, Carlsbad, CA). Normalized threshold cycle data from the real-time instrument were calculated and interpreted using the PCR Array Data Analysis software tool (SABiosciences) which utilizes the ΔΔC_T_ method of data analysis and normalizes all results to five housekeeping genes. The relative expression of each gene in either the TI or TII cells of the LPS-treated animals was compared with the expression in the TI or TII cells of control animals; significance was assessed by an unpaired Student's *t*-test, with a *p* value of ≤0.05 considered statistically significant. Validation of expression for select genes was accomplished using quantitative real-time PCR with gene expression assays purchased from Applied Biosystems. Relative amounts of mRNA for the validation studies were calculated using the comparative threshold method with 18S RNA as the internal control. Significance was again assessed by an unpaired Student's *t*-test, with a *p* value of ≤0.05 being considered significant.

### Western blot analysis

After instillation of LPS or saline vehicle (for uninjured control) into the lungs of rats, TI and TII cells were isolated via FACS 18 hours afterwards as described above. Protein lysates from injured and uninjured TI and TII cells were studied using standard Western blotting techniques. Antibodies used were CCR5, SPP1 (Abcam, Cambridge, MA) and β-actin (Sigma). Densitometric quantitation was performed using ImageJ software (www.NIH.gov).

### Immunohistochemistry

Immunohistochemistry was performed on cytocentrifuged preparations of mixed rat lung cells. Adult Sprague-Dawley rats were euthanized with an intraperitoneal injection of pentobarbital (100 mg/kg) and the lungs were removed, digested with porcine grade IV elastase (Sigma) and minced and then filtered to form a single cell suspension, from which aliquots were taken and spun down onto slides. These cytocentrifuged mixed lung cell preparations were then fixed in 4% paraformaldehyde prior to immunohistochemical staining. Antibodies used were phospho-IKKα/β (Cell Signaling Technologies, Danvers, MA), RTI40 and RTII70. Secondary antibodies were conjugated to Alexa Fluor 488 or 594 (Invitrogen). Control slides utilized a non-specific IgG primary antibody. All slides were mounted with Prolong Antifade Reagent (Invitrogen) and images were captured with a Leica DC500 camera on a Leica Orthoplan microscope.

### Measurement of cytokines via Enzyme-Linked Immunosorbent Assay (ELISA)

Cell culture supernatants were assayed using ELISA for TNF-α, IL-6 (BD Biosciences, San Jose, CA) or IL-1β (R&D Systems, Minneapolis, MN) per manufacturer's instructions.

### Surfactant isolation

Adult Sprague Dawley rats were euthanized as described above. Bronchoalveolar lavage was collected by first instilling 3 ml of 10 mM PBS with Ca^2+^ and Mg^2+^ and then flushing the solution back and forth into the lungs three times. The procedure was repeated with another 3 ml three additional times and the BAL from a single animal was pooled. The BAL was centrifuged at 500 g for 5 minutes to remove cells and cellular debris. The supernatant was collected and protease inhibitors (Sigma) were added prior to centrifugation at 27,000 g for 60 minutes. The pellet, containing large aggregate surfactant, was collected and resuspended in surfactant buffer (10 mM Tris, 154 mM NaCl, 1.5 mM CaCl_2_, pH 7.4) [20]. An aliquot was extracted and assayed for total protein and phospholipid content (kindly performed by Cheri Chapin, UCSF). Surfactant was added to cells in culture; for studies involving the study of surfactant and LPS, surfactant was added 2 hours prior to the addition of LPS.

### Statistical Analysis

All experiments were performed a minimum of three times unless otherwise stated. For comparisons between the control and treated groups, unpaired Student's *t*-test was employed. For comparisons of multiple groups, a one-way analysis of variance (ANOVA) followed by post-hoc analysis using the Bonferroni *t*-test was conducted using GraphPad Prism 5.0 software (GraphPad, San Diego, CA). A *p* value of <0.05 was considered significant.

## Results

### Differential cytokine expression between TI and TII cells

**Figure 1:**
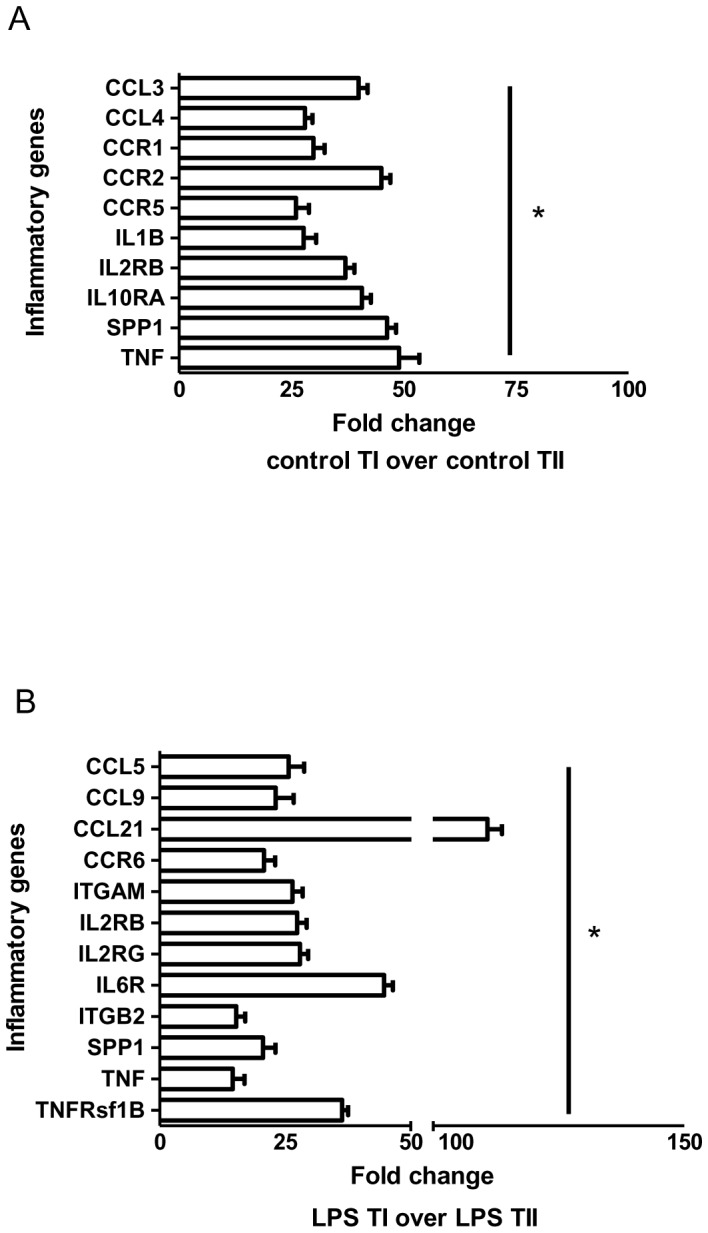
Expression of inflammatory mediators in TI and TII cells. RT^2^ Profiler Array for rat inflammatory cytokines and receptors was performed with mRNA from TI and TII cells isolated from control and LPS-injured rats (LPS 10 mg/kg). Results are expressed as fold changes of the various genes as illustrated of control TI cells over control TII cells (Figure 1A) and of LPS-stimulated TI cells over LPS-stimulated TII cells (Figure 1B). The top 10 differentially expressed genes are listed for control TI versus TII cells and the top 12 are listed for LPS-stimulated TI versus TII cells. For each condition, n = 6 and *p<0.05.

Inflammatory gene expression analysis in rat TI and TII cells was performed using a PCR array that assayed the expression of 84 genes affiliated with inflammation. We considered a gene to be significantly differentially regulated if the difference in expression between the cell types was >2-fold and the corresponding *p* value was <0.05. TI cells from control uninjured rats demonstrated a statistically significant higher level of expression of multiple genes (43 of 84 genes assayed) when compared to control uninjured TII cells, with CCL3, CCL4, CCR1, CCR2, CCR5, IL10ra, IL1β, IL2rb, SPP1 (secreted phosphoprotein 1 or osteopontin) and TNF being the ten most differentially expressed (Figure 1A). TI cells from LPS-injured animals demonstrated enhanced induction in 50 of the 84 genes assayed when compared to TII cells from the same injured animals, with CCL21, CCL9, CCL5, CCR6, IL2rb, IL2rg, IL6r, ITGAM (Integrin, alpha M), ITGB2 (Integrin, beta 2), SPP1, TNF and TNFrsf1b (TNF receptor superfamily, member 1b) exhibiting the most differential expression (Figure 1B). When comparing TI cells from control animals to TI cells from LPS-injured animals, despite the trend towards higher expression in TI cells of nearly 1/3 of the genes from LPS-treated animals, only three genes exhibited >2-fold increase that was statistically significant: Casp1, CCL19 and CXCL4. In TII cells, only five genes – CCL19, CRP, IL13, IL18 and LTA (lymphotoxin alpha or TNF-β) – were significantly up-regulated in LPS- treated animals compared to controls.

**Figure 2:**
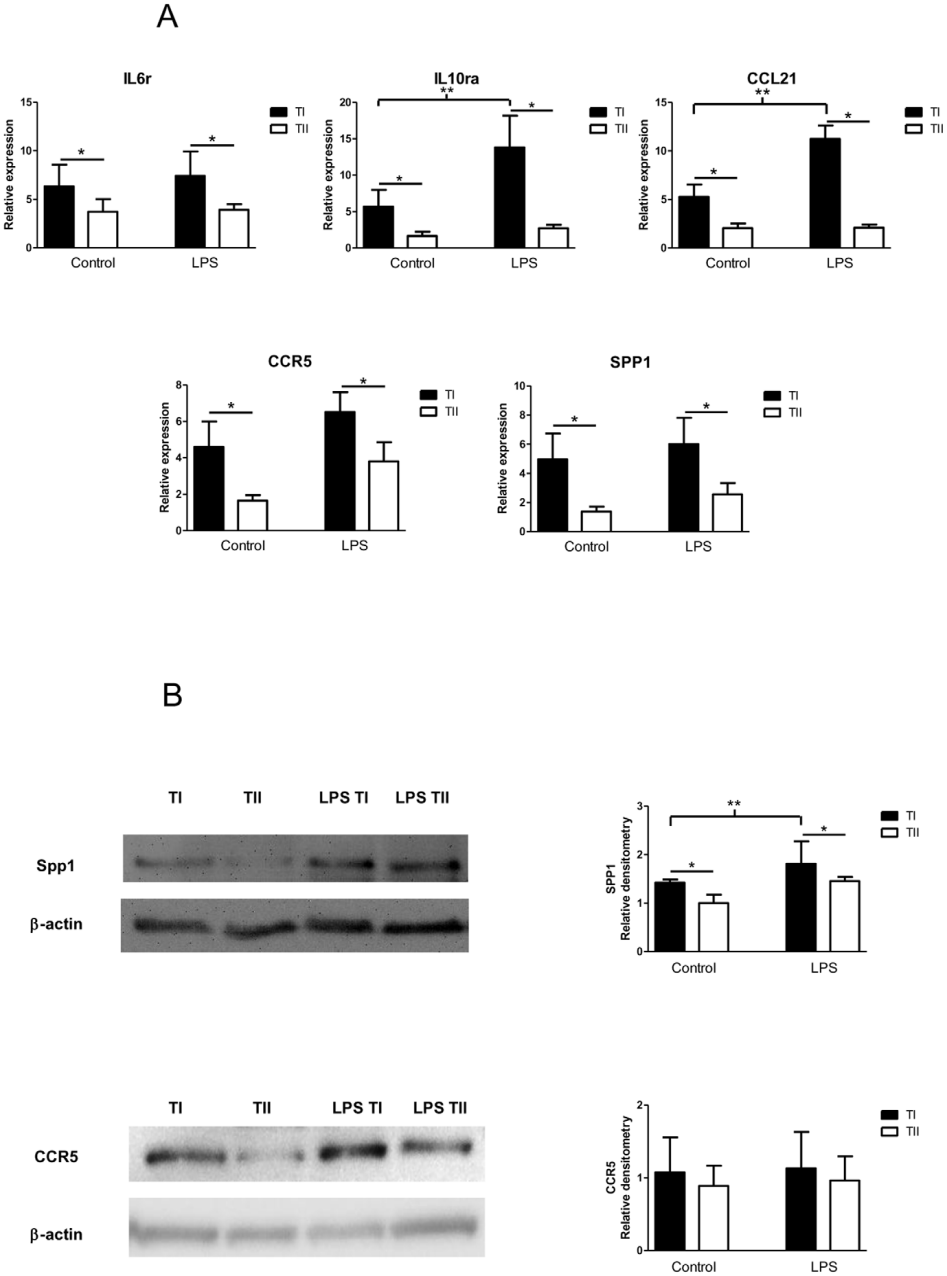
Validating enhanced expression of inflammatory mediators. A. qPCR assay: IL6r, IL10ra, CCL21, CCR5 and SPP1 expression in TI and TII cells isolated from control (n = 4) and LPS-injured (LPS 10 mg/kg) animals (n = 4) was measured using qPCR employing TaqMan probes and primers. Relative to the expression of the above genes in TII cells of control animals, uninjured TI cells had higher levels of expression of all inflammatory mediators studied. LPS injury increased cytokine transcripts of all genes compared to controls for TI and TII cells, but the only in the case of IL10ra and CCL21 in injured TI cells was the fold-increase significant over uninjured TI cells (*p<0.05). Data is expressed as fold expression of inflammatory mediator transcript over TII cell controls ± SEM; all values were normalized to 18S. B. Western blot analysis: Western blots of cell lysates from TI and TII cells of control and LPS-injured (LPS 10 mg/kg) rats were probed with antibodies against CCR5 and SPP1. β-actin staining is shown to exhibit equalization of protein loading. Blots represent 3 separate experiments. Densitometry measurements suggest that control TI cells contain ∼20% more SPP1 than control TII cells, and injured TI cells contain ∼30% more SPP1 protein than control TI cells. Both differences are statistically significant. Furthermore, SPP1 protein expression from injured TI cells was significantly greater than from control TI cells. Control TI cells contain only ∼5% more CCR5 than control TII cells and injured TI cells express ∼15% more CCR5 than injured TII cells, but the differences were not statistically significant. (*p<0.05).

We selected five genes that had well-studied TaqMan qPCR probes and primers available to validate our PCR array results from our comparison of control TI and TII cells and of LPS-stimulated TI and TII cells - CCL21, CCR5, IL6r, IL10ra and SPP1. We chose to study CCL21, a C-C chemokine that is chemotactic for thymocytes and activated T-cells in vitro, and IL6r because they were the two most differentially expressed genes of all assayed. We selected to validate SPP1 and CCR5, as they are well-studied inflammatory mediators (see below), and IL10ra, interleukin 10 receptor alpha, because its ligand, IL-10, is considered an anti-inflammatory cytokine [21]. For each gene studied, both control and LPS-treated TI cells exhibited greater-fold expression than control or LPS-treated TII cells that was statistically significant (Figure 2A), which is consistent with our PCR array results. In the cases of IL10ra and CCL21, the relative expression levels in LPS-treated TI cells was significantly greater than in control TI cells (p<0.05). LPS-injured TI cells exhibited >2-fold induction of both IL10ra and CCL21 in our PCR array, but failed to reach statistical significance when compared to expression in control TI cells.

Since many transcripts undergo post-transcriptional modification, we examined whether our mRNA data translated into similar differences in protein expression. We chose to measure the protein expression of SPP1 and CCR5 as well-characterized antibodies were available for these proteins. SPP1 is an important participant in both innate and acquired immunity [22], acting as a chemotactic factor for macrophages, dendritic cells and T cells [23], [24], and is strongly expressed in the alveolar macrophages of patients with ARDS [24], [25]. SPP1 also acts as an important signal transducer that interacts with different pathways to mediate immune responses. SPP1 can activate NF-κB transcription, which promotes cytokine and inflammatory mediator expression [23] either by signaling through the Src and FAK tyrosine kinases to activate NF-κB, or by phosphorylating IκBα, an inhibitor of NF-κB, which induces IκBα degradation and subsequent translocation of NF-κB to the nucleus. Our qPCR data validated the finding of high levels of SPP1 in LPS-injured TI cells. Western blotting of lysates from both control and LPS-stimulated alveolar epithelial cells demonstrated that TI cells contain more SPP1 than their TII cell counterparts, and that injured TI cells had significantly more SPP1 protein than either injured TII or control TI or TII cells. C-C chemokine receptor 5 (CCR5), a member of the beta chemokine receptor family, functions as a receptor for many C-C chemokines including monocyte chemoattractant protein 2 (MCP-2), macrophage inflammatory protein-1α and -1β (MIP-1α, MIP-1β), and regulated on activation normal T expressed and secreted protein (RANTES) [26]. CCR5 is also a co-receptor for HIV-1 and the target for the HIV drug Maraviroc [27]. Our qPCR validation data indicate that CCR5 is more highly expressed in control TI cells versus TII cells and in LPS-treated TI versus TII cells (Figure 2A), however, the protein expression levels of CCR5 were not statistically different among the cell types studied (Figure 2B), suggesting that CCR5 in alveolar epithelial cells may undergo more post-transcriptional modification than SPP1.

We anticipated that a higher number of genes in either LPS-injured TI or TII cells would have been up-regulated compared to their non-injured controls, given that prior data from our lab demonstrated that TI cells in culture produce significantly more TNF-α, IL-6 and IL-1β via ELISA analysis than controls after LPS stimulation [9]. However, when isolating TI or TII cells from LPS-injured rat lungs, we realize that we are actually studying alveolar cells that have been exposed to their inflammatory milieu, including macrophages, endothelia, surfactant, immune cells, plasma factors, etc. In order to separate cell-specific responses to LPS stimulation alone, we moved to an *in vitro* model.

### Cultured TI cells produce more TNF-α, IL-6 and IL-1β than cultured TII cells

**Figure 3:**
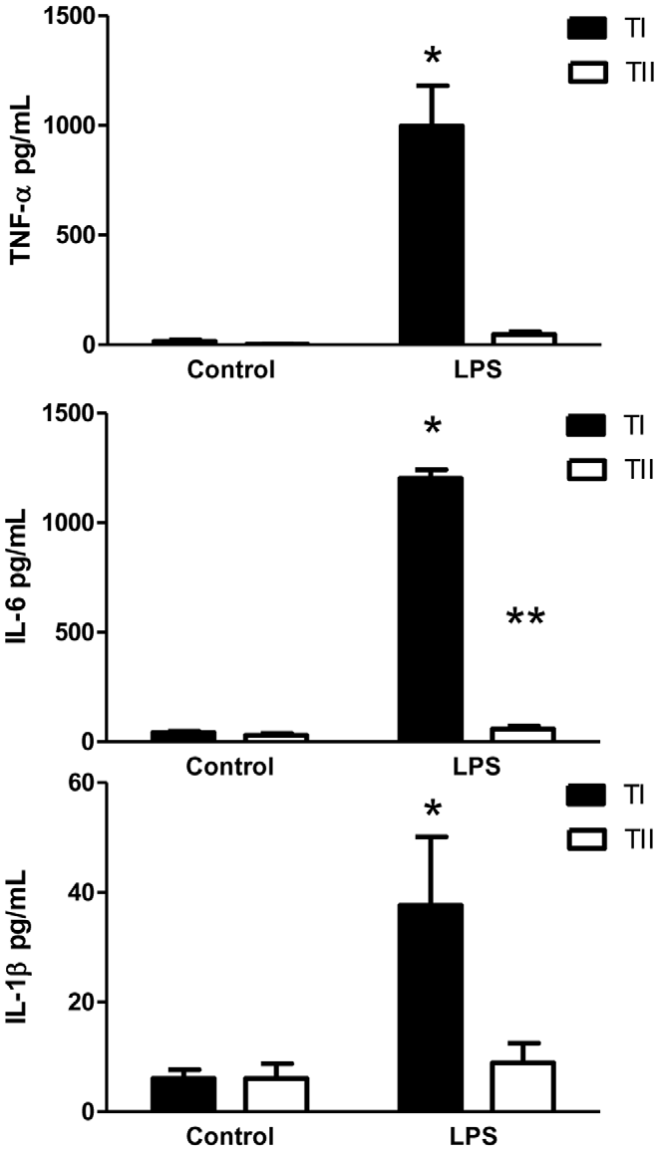
Cytokine response in cultured TI and TII cells stimulated with LPS. TNF-α, IL-6 and IL-1β were detected in the supernatants of cultured TI and TII cells isolated via FACS both at baseline and after *in vitro* LPS (10 µg/ml) stimulation. TNF-α, IL-6 and IL-1β production in LPS-stimulated cells was significantly increased over baseline for TI cells (n = 5 for each cell type, *p<0.05). Levels of IL-6 in LPS-stimulated TII cells were significantly greater than in control TII cells. Results are expressed as mean level of cytokine ± SEM; *p <0.05 for control TI vs. LPS-treated TI cells, **p<0.05 for TII control vs. LPS-treated TII cells.

TII cells are known to possess immunomodulatory functions, including the ability to produce various cytokines upon stimulation [3], [10]–[12]. Both TI and TII cells were isolated from the lungs of rats via FACS and cultured overnight to allow recovery from the isolation procedure prior to the addition of LPS 10 µg/ml. Eighteen hours later, the media was collected and assayed for cytokine content via ELISA. At baseline, cultured TI cells produced slightly more TNF-α (15.8±5.8 pg/ml) and IL-6 (41.6±6.6 pg/ml) than their TII cell counterparts (TNF-α 1.9±0.7 pg/ml; IL-6 37.7±8.0), while levels of IL-1β were nearly equivalent for both cell types. However, after LPS stimulation, TI cells produced significantly more TNF-α (997.2±183.9), IL-6 (1201.9±40.6) and IL-1β (37.7±12.4) than TII cells (TNF-α 47.4±12.1; IL-6 53.9±14.1; IL-1β 8.9±3.6; *p<0.05) (Figure 3). LPS did increase cytokine production in TII cells over baseline, but only in the case of IL-6 was statistical significance reached (**p<0.05).

### TII cells produce less cytokines than expected after LPS treatment

**Figure 4:**
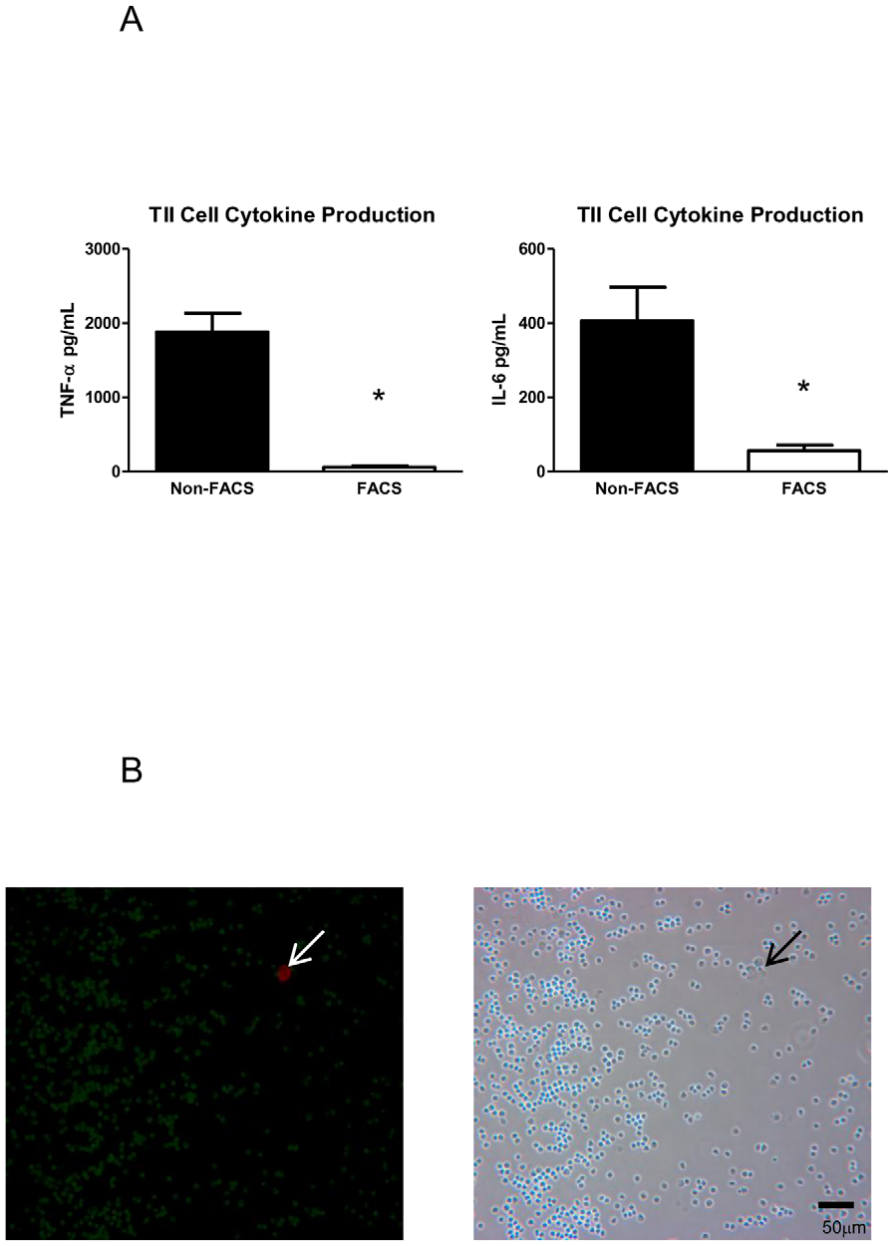
FACS TII cells. A. Cytokine expression in non-FACS TII cells is greater than in FACS TII cells. TNF-α and IL-6 levels were higher in TII cells isolated in the conventional manner (non-FACS) than in FACS-isolated TII cells after treatment with LPS (10 µg/ml). TNF-α levels in FACS TII cells were 97% lower than in non-FACS TII cells (n = 7, *p<0.05). Similarly, IL-6 levels in FACS TII cells were 77% lower than in non-FACS TII cells (n = 6, *p<0.05). Results are expressed as mean level of cytokine ± SEM. B. Immunohistochemistry of FACS TII cells. A cytocentrifuged preparation of FACS TII cells was double-stained with a marker for TII cells, RTII70 (green), and a marker for TI cells, RTI40 (red). The white arrow points to the lone TI cell in the field. The corresponding phase contrast image is shown. Magnification is 20×.

Recent reports of TNF-α levels in primary TII cell cultures range from ∼800 to 1000 pg/ml 24 hours after LPS stimulation at doses ranging from 0.01 to 10 µg/ml [10], [11], [28], [29]. The levels of cytokine expression in our FACS-isolated cultured TII cells were notably less (Figure 3). The most obvious difference between our findings and those published reports was that our TII cells were isolated via FACS. In order to determine the cause of these seemingly discordant results, we isolated TII cells utilizing a standard published protocol for TII cell isolation [30] and treated the TII cells in the exact same fashion as our FACS-isolated TII cells: we placed the cells in culture in media with fetal bovine serum overnight, stimulated the cells with LPS in serum-free media the next morning, and collected the cells and supernatant 18 hours later. We chose to measure TNF-α and IL-6 secretion via ELISA as the overall level of IL-1β production for both cell types was low and alterations in levels of expression would be difficult to appreciate. Both at baseline and after LPS stimulation, TII cells isolated in the conventional manner (non-FACS) demonstrated much higher levels of TNF-α (1882.4±255.4 pg/ml) and IL-6 (406.3±84.5 pg/ml) than TII cells isolated by FACS (TNF-α 59.0±19.8 pg/ml; IL-6 56.2±16.5 pg/ml) (Figure 4A). The numbers for the non-FACS TII cells were more consistent with published reports of LPS-induced cytokine production in TII cells, suggesting that the difference in cytokine production may be due in part to the contaminating macrophages present in each preparation.

### Alveolar macrophages stimulate TII cell cytokine production in the presence of LPS

**Figure 5:**
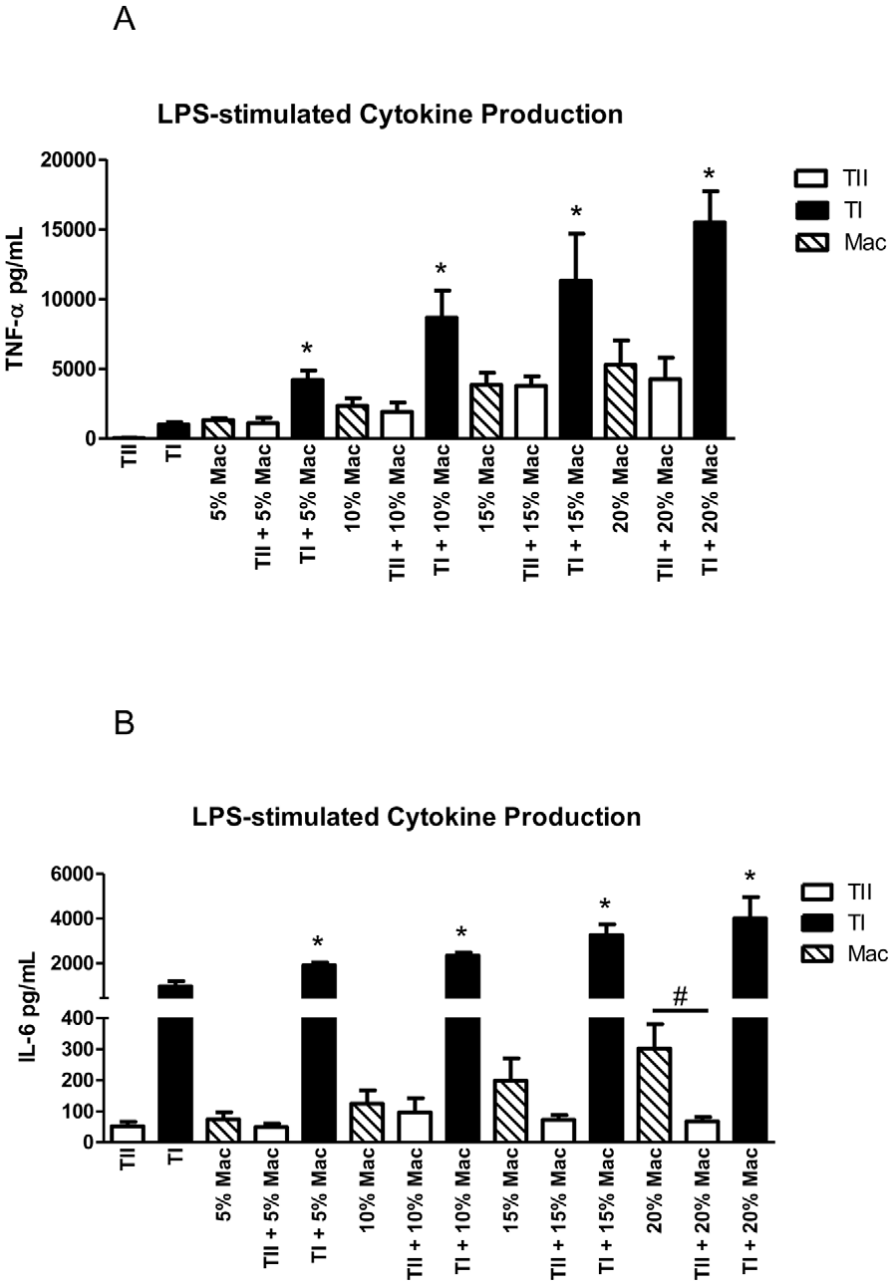
Cytokine production in LPS-stimulated macrophages and alveolar epithelial cells. TNF-α (Figure 5A) or IL-6 (Figure 5B) cytokine expression in TI cells co-cultured with progressively higher concentrations of macrophages significantly increased TNF-α and IL-6 cytokine production in a dose-dependent manner after stimulation with LPS (10 µg/ml) (n = 4 for each set of experiments). Addition of macrophages to TII cells did not significantly alter TNF-α or IL-6 production from results obtained examining LPS-treated macrophages alone, except in TII cells co-cultured with 20% macrophages, where IL-6 levels were significantly lower. Results are expressed as mean level of cytokine in pg/ml ± SEM, *p<0.05.

The conventional method of rat TII cell isolation typically produces TII cell purities that are reported to be between 85–97%, resulting in a 3–15% contamination rate, with macrophages most often implicated as the contaminating cell type [12], [31], [32]. With FACS, we were able to achieve TII cell purities between 95–98% (Figure 4B), resulting in a 2–5% rate of contamination. To determine if contaminating macrophages could account for enhanced cytokine expression in non-FACS cultured TII cells, we isolated macrophages and added them to our FACS TII cells in culture at varying concentrations. The numbers of macrophages were chosen to reflect similar degrees of contamination of TII cell preparations. The percentage of macrophages was calculated from the number of alveolar epithelial cells used in the experiments (500,000 cells/well), as previously described in Methods. When stimulated with LPS, increasing the concentrations of macrophages produced increasing amounts of TNF-α (5% Mac: 1328.4±133.3; 10% Mac: 2341.6±551.4 pg/ml, 15% Mac: 3834.5±875.4 pg/ml, 20% Mac: 6021.0±1041.3 pg/ml) and IL-6 (5% Mac: 73.7±23.3 pg/ml, 10% Mac: 124.4±43.4 pg/ml, 15% Mac: 198.8±71.6 pg/ml, 20% Mac: 302.4±67.9 pg/ml) as expected. We then co-cultured FACS-isolated TII cells with 0%–20% macrophages and treated the cells with LPS prior to measuring supernatant cytokine expression. We discovered that increasing the number of macrophages predictably increased, in a dose-dependent fashion, TNF-α production (TII+0% Mac: 47.3±12.1 pg/ml; TII+5% Mac: 1090.3±402.3 pg/ml; TII+10% Mac: 1910.6±658.3 pg/ml; TII+15% Mac: 3770.4±688.1 pg/ml; TII+20% Mac: 4137.2±1559.9 pg/ml) to levels that have been reported in the literature for TII cells stimulated with LPS [11], [28]. Co-culturing FACS-isolated TII cells with 5–20% macrophages increased IL-6 production over LPS-stimulated TII cells alone, but the levels were not statistically different (TII+0% Mac: 53.9±14.1 pg/ml; TII+5% Mac: 48.9±11.5 pg/ml; TII+10% Mac: 95.9±46.4 pg/ml; TII+15% Mac: 72.7±15.0 pg/ml; TII+20% Mac: 67.3±13.5 pg/ml) (n = 3, Figure 5B). However, IL-6 levels from stimulated co-cultures of TII cells and 20% macrophages was significantly reduced over levels for stimulated macrophages alone (#p<0.05).

### Alveolar macrophages significantly increase TI cell cytokine production in the presence of LPS

The addition of macrophages to TII cells resulted in an increase in cytokine production after treatment with LPS, albeit to levels less than that for macrophages alone. Given that TI cells also share a microenvironment with macrophages, we performed similar experiments with TI cells. The addition of escalating numbers of macrophages to cultured TI cells prior to stimulation of the co-culture with LPS resulted in a dramatic dose-dependent increase in TNF-α (TI+0% Mac: 997.2±183.9 pg/ml; TI+5% Mac: 4198.5±680.5 pg/ml; TI+10% Mac: 8653.4±1957.6 pg/ml; TI+15% Mac: 11303.4±2749.0 pg/ml; TI+20% Mac: 15492.4±2238.8 pg/ml) and IL-6 (TI+0% Mac: 1201.9±40.6 pg/ml; TI+5% Mac: 1911.1±131.3 pg/ml; TI+10% Mac: 2345.9±138.2 pg/ml; TI+15% Mac: 3265.2±78.1 pg/ml; TI+20% Mac: 4013.4±953.2 pg/ml) expression that was significantly greater than the addition of the same number of macrophages to cultured FACS-isolated TII cells. These results not only reinforce our findings that TI cells produce more cytokines per cell than TII cells both at baseline and upon LPS exposure, but also reveal that macrophages have a more profound effect on TNF-α and IL-6 secretion on TI cells than TII cells in response to LPS (Figure 5A & B). Furthermore, LPS stimulation of a co-culture of macrophages and TI cells produced significantly more TNF-α and IL-6 than LPS stimulation of macrophages alone, suggesting that interplay between macrophages and TI cells clearly enhances immune system activation.

### Conditioned media from LPS-stimulated macrophages increases baseline TI cell cytokine expression

**Figure 6:**
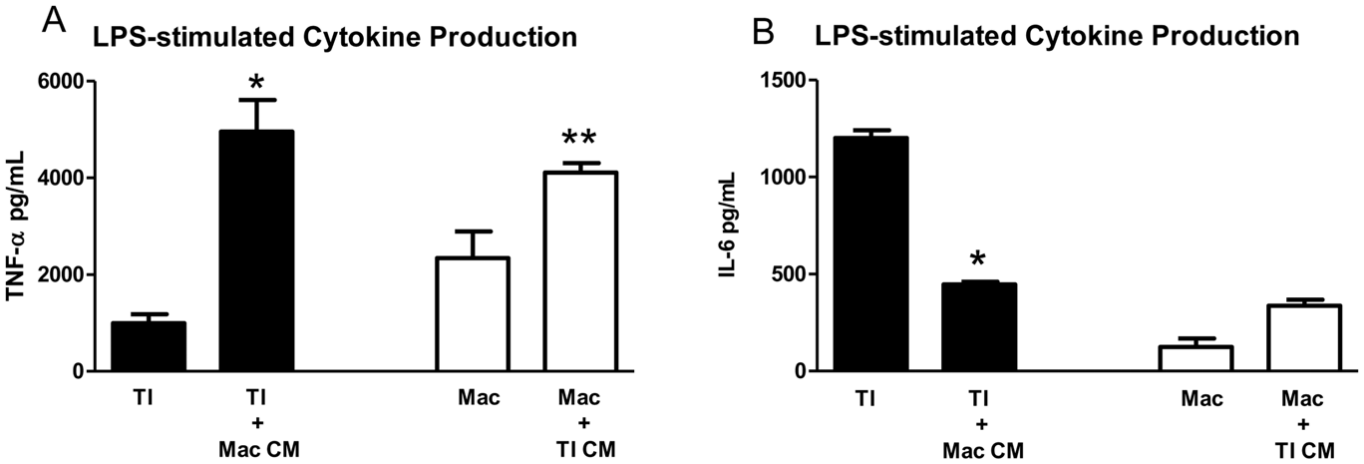
Effect of conditioned media on TI cell and macrophage cytokine production. Conditioned media from LPS-stimulated macrophages (Mac CM, n = 4, LPS 10 µg/ml) significantly increased TI cell TNF-α production but decreased IL-6 production when compared to TI cells stimulated with LPS alone (n = 6, *p<0.05). Addition of conditioned media from LPS-stimulated TI cells (TI CM, n = 4, LPS 10 µg/ml) to macrophages increased both TNF-α and IL-6 expression compared to macrophages stimulated with LPS alone (*p<0.05 for TNF-α). Results are expressed as mean level of cytokine in pg/ml ± SEM.

Soluble factors present in conditioned media could be one explanation for the sizeable cytokine response of TI cells co-cultured with macrophages prior to LPS stimulation. In an attempt to gain insight into how macrophages may promote such a heightened cytokine response in TI cells, we collected the supernatant from cultured macrophages (10% Mac) that had been stimulated with LPS and then added the conditioned media to cultured TI cells. Conditioned media from LPS-stimulated macrophages (10% Mac) increased TI cell TNF-α production (4111.2±195.6 pg/ml), but not nearly to the levels seen after LPS addition to co-cultures of TI cells and macrophages. This same macrophage conditioned media, however, blunted the degree of TI cell IL-6 production (337.0±31.2 pg/ml) when compared to LPS-treated TI cell IL-6 expression, which is in contrast to the high levels of IL-6 measured in LPS-stimulated co-cultures of TI cells and macrophages. Conditioned media from LPS-stimulated macrophages did not significantly increase TII cell cytokine production over TII cells stimulated with LPS, nor did conditioned media from LPS-stimulated TII cells alter macrophage cytokine production over LPS treatment alone (results not shown). The addition of conditioned media from LPS-stimulated TI cells to cultured macrophages did increase TNF-α (4956.7±654.1 pg/ml) and IL-6 (446.3±13.8 pg/ml) levels above those seen with the addition of LPS to macrophages (10% Macs) alone (Figure 6), but again, less than the levels seen with the co-culture of TI cells and macrophages after LPS treatment (Figure 5). These results suggest that while substances secreted by activated macrophages can stimulate TI cell production of TNF-α and inhibit TI cell production of IL-6, cell-cell interaction between macrophages and TI cells significantly enhances the cytokine production of both TNF-α and IL-6, additionally overcoming the inhibition of TI cell IL-6 production by activated macrophage conditioned media.

### Exogenous surfactant decreases LPS-stimulated TI cell cytokine production

**Figure 7:**
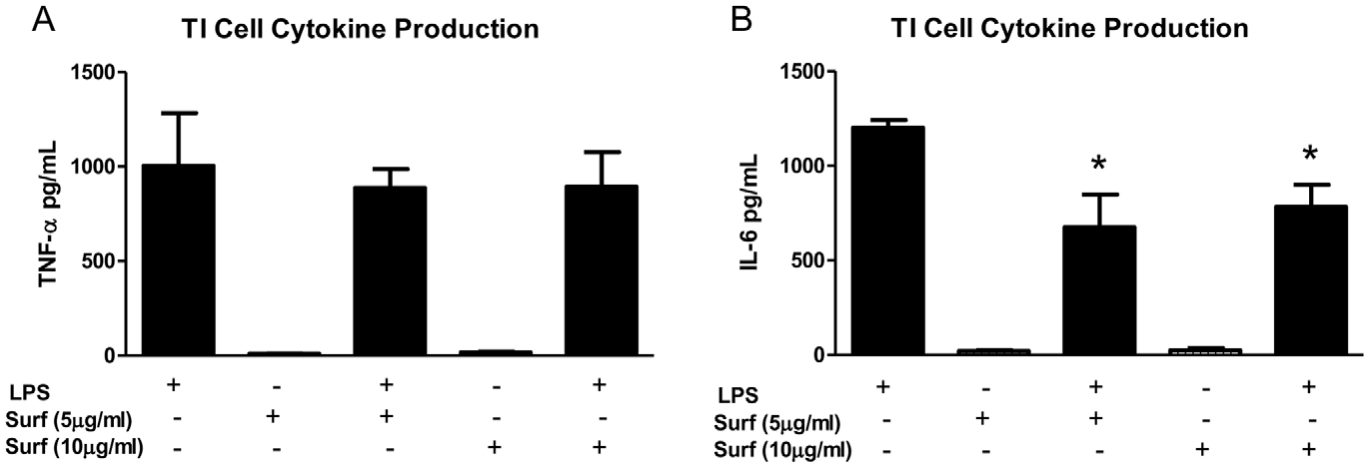
Effect of surfactant on LPS-stimulated cytokine response in TI cells. Surfactant significantly decreased LPS-induced IL-6 production in TI cells by 43% with 5 µg/ml surfactant (n = 4) and 35% with 10 µg/ml surfactant (n = 3) when compared to IL-6 production in TI cells stimulated with LPS alone (LPS 10 µg/ml, n = 4, *p<0.05). Surfactant also decreased the level of TNF-α production in TI cells stimulated with LPS by ∼11% at both concentrations, but the reduction was not significant. Surfactant alone did not alter TNF-α or IL-6 expression in the absence of LPS (n = 3).

It has been long reported that surfactant can decrease the degree of lung inflammation in response to endotoxin stimulation [33]–[35]. These studies, coupled with our results revealing a decrease in IL-6 production in co-cultures of TII cells and macrophages treated with LPS, prompted us to investigate the role of surfactant in cytokine expression in both TI and TII cells. The addition of exogenous rat surfactant (5 or 10 µg/ml) to cultured TI cells did not alter cytokine production at baseline, but significantly blunted the cytokine response of TI cells to LPS for IL-6 (LPS: 1201.9±40.6 pg/ml; LPS +5 µg/ml surfactant: 823.6±102.4 pg/ml; LPS +10 µg/ml surfactant: 782.8±71.1 pg/ml; *p<0.05). Levels for TNF-α in TI cells treated with surfactant and LPS (LPS +5 µg/ml surfactant: 886.8±99.1 pg/ml; LPS +10 µg/ml surfactant: 892.7±142.6 pg/ml) were not significantly different than TI cells stimulated with LPS alone (1256.3±259.2 pg/ml)(Figure 7). The addition of exogenous rat surfactant (5 or 10 µg/ml) to TII cells did not alter cytokine production in response to LPS (data not shown).

### LPS-induced cytokine response in TI cells may be due to stimulation of the NF-κB/IκB pathway

**Figure 8:**
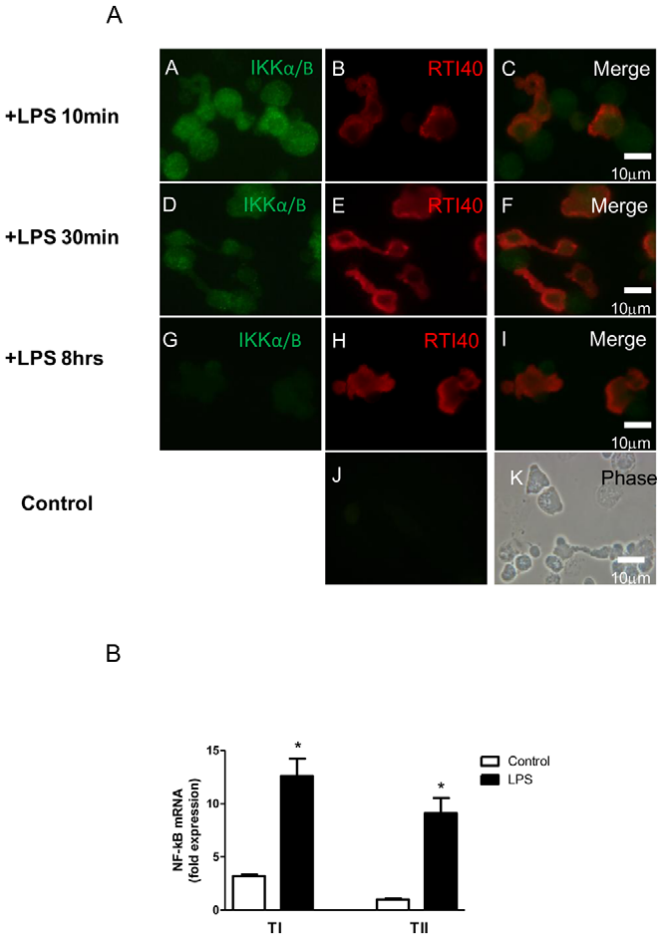
LPS may activate cytokines in TI cells via the NF-kB/IκB pathway. A. LPS stimulates IKKα/β expression in TI cells. Cytocentrifuged mixed cell preparations from LPS-treated (10 µg/ml) TI cells were double-stained with an antibody to phospho-IKKα/β and RTI40, a TI cell marker. A, D, G) Mixed lung cell preparation stained with phospho-IKKα/β at various timepoints after LPS stimulation, demonstrating decreasing staining intensity of phospho-IKKα/β over time; B, E, H) cells stained with RTI40 to display TI cells; C, F, I) merged images demonstrating colocalization of phospho-IKKα/β and a TI cell-specific marker. J) Control image showing lack of staining in the absence of a specific primary antibody; K) phase contrast image of the control slide. Image magnification is 40×. B. LPS induces NF-κB transcript in cultured TI cells. Expression of NF-κB transcript in cultured TI cells increased after LPS stimulation (LPS 10 µg/ml). Relative to the amount of transcript found in TI cells at baseline (n = 4), LPS injury increased NF-κB levels by 9-fold in TI cells (n = 4, *p<0.05). LPS also increased NF-κB transcripts 8-fold in TII cells (n = 5) over TII controls (n = 5, ** p<0.05). Data is expressed as fold expression of NF-κB mRNA over control ± SEM and all values were normalized to 18S.

The NF-κB/IκB pathway has been implicated in the inflammatory cascade caused by a variety of stimuli, including LPS, in TII cells [29], [36]–[38]. LPS, acting through the toll-like receptor 4 (TLR4), can activate the phosphorylation of IKKα/β, which allows phosphorylation of IκB within the NF-κB complex, leading to ubiquitination of the IκB complex and release of NF-κB, allowing its translocation to the nucleus where it enhances transcription of various inflammatory mediators. Whether this mechanism of inflammation was present in TI cells was unclear. Using immunohistochemistry, phospho-IKKα/β staining was present in cultured TI cells as early as 10 minutes after LPS stimulation, with a progressive reduction in staining intensity at 30 minutes, 1 hour, 4 hours, 8 hours and 18 hours (Figure 8A). Similar staining was seen in TII cells (data not shown). NF-κB expression, as measured by qPCR in cultured cells, is elevated in both TI and TII cells after LPS stimulation, suggesting that LPS may act via the well-described NF-κB/IκB pathway in both cell types to stimulate cytokine secretion (Figure 8B).

## Discussion

The alveolar epithelium has a number of very important functions, primary among them gas exchange and existence as a tight barrier to separate organism from environment. Alveolar TII cells have traditionally been considered the immune cell of the alveolar epithelium as this cell produces surfactant, cytokines and chemokines, all of which have been implicated as being important factors in lung immunity. TII cells, however, only cover approximately 5% of the internal surface area of the lung, while TI cells cover the remaining 95% [8], prompting the question of whether TI cells could participate in the innate immune response since they comprise the majority of the epithelial barrier. Earlier, Mason and colleagues had studied TII cells that had been cultured until they exhibited phenotypic characteristics similar to TI cells (TI-like cells) and demonstrated that after LPS or viral stimulation, these cells could produce CINC-2, CINC-3, LIX, MIP-2 and MCP-1 [39], [40], suggesting the possibility that TI cells could play a role in innate immunity. Prior methods of TI cell isolation [41] involved labeling the cells with magnetic beads and performing both positive and negative sorting of the cells over magnetic columns, which resulted in TI cell purities of 75–90% [42], [43]. But as techniques became available to isolate relatively pure populations of TI cells via FACS, we employed such methods to study primary alveolar TI cells. We were able to recently report that TI cells are capable of producing TNF-α, IL-6 and IL-1β when exposed to LPS [9], advocating a role for TI cells in the innate immune response in the lung. Our current study reports not only that TI cells are able to produce a wide range of cytokines, chemokines, and other mediators of inflammation, but that there is differential expression of a number of these mediators between TI and TII cells, suggesting different roles for these cell types in the inflammatory response of the lung.

To gain a broader view of the potential for TI cells to participate in lung inflammation, we performed PCR array analysis comparing control and LPS-stimulated TI and TII cells. There was a large number of inflammatory mediators more highly expressed in TI cells compared to TII cells at baseline, with 43 of the 84 genes demonstrating fold-increases >2 that were statistically significant. Similar widespread gene induction was seen in LPS-injured TI cells, with TI cells having much higher-fold gene expression than TII cells from the same injured animals. With >50% of the genes assayed exhibiting higher expression in TI cells from either control or LPS-injured animals when compared to TII cells, we expected a larger number of genes to be up-regulated upon comparison between either control TI and LPS-treated TI cells or control TII and LPS-treated TII cells. When comparing control and LPS-treated TI cells, only three genes were significantly induced in LPS-treated TI cells versus controls. Similarly, in our comparison of control and LPS-treated TII cells, only five genes were more highly expressed in LPS-treated TII cells than controls. The relatively high expression levels of the majority of the genes at baseline in both sets of control cells may have rendered the up-regulation of genes after LPS treatment less striking by comparison, leaving us to underestimate the true number of mediators that may have been enhanced by injury.

Cell isolation, in and of itself, can trigger the immune response by promoting cellular stress during the processes of enzymatic digestion, tissue mincing, and filtering. Studies have shown that TNF-α production from freshly isolated rat alveolar epithelial cells can decrease dramatically, from 1200 pg/ml immediately after isolation to 50 pg/ml 48 hours later [12]. Subjecting the cells further to FACS may have contributed to the degree of stress experienced by the cells. Additionally, alveolar epithelial cells isolated from LPS-treated rats have not only been exposed to the intratracheally instilled LPS, but also to growth factors, surfactant, immune cells, endothelial cells, cytokines, chemokines and a host of other proteins released by surrounding cells in response to LPS that end up in the alveolar microenvironment. These products of inflammation undoubtedly affect cytokine expression in both TI and TII cells. Taken together, these findings suggest that the cell isolation procedure and cell sorting may have resulted in the production of higher levels of inflammatory mediators at baseline in control cells that may not have been present if the cells could be studied *in vivo*. Such results led us to use cultured primary cells that had been given at least 24 hours to recover from the isolation process before stimulation with LPS or other agents. Furthermore, this method allowed the assay of cytokine secretion via ELISA analysis of the conditioned media. Despite the fact that working with cells from LPS-injured animals is the more valid *in vivo* model, we used *in vitro* studies of cultured cells to isolate the specific responses of each cell type to various agents and conditions.

We were very surprised to find that primary cultured FACS TII cells produced significantly lower levels of selected cytokines than previously published reports for LPS-stimulated TII cells [11], [28], which is ∼10–30% of the cytokines liberated by primary cultured FACS TI cells exposed to identical amounts of LPS for the same periods of time. This finding is noteworthy, since the TII cell has long been considered to be the immune effector cell of the alveolar epithelium [1], [44]. It can be argued that the mere act of cell isolation, not to mention the process of FACS, may have rendered TII cells less capable of cytokine production. However, FACS-isolated TI cells were able to produce significantly more cytokines than TII cells both at baseline and upon LPS treatment.

One may contend that our method of comparison of cytokine levels in cultured TI and TII cells, plating by cell number, may not be the ideal manner in which to contrast the inflammatory responses of the two cell types, and that the greater surface area of TI cells may explain why they produce more cytokines. The surface area ratio of TI∶TII cells is 43∶1 [8] and the ratio of numbers of TI∶TII cells in the lung is 1∶2 [45], so in this instance, even if TII cells were to produce 43 times the amount of cytokines after LPS treatment, their levels of TNF-α and IL-6 production would still be less than that of TI cells. Alternative methods of culturing cells based on protein or DNA content or surface area ratios were considered, but have inherent problems as well, especially since the biologically significant ratio of TI to TII cells when comparing cell function is not known. The desire for consistency and reproducibility within our experiments, particularly when dealing with such a small number of TI cells obtained per isolation (∼1×10^6^ cells/rat) and the need to standardize our co-culture experiments led to our use of cell number in plating our cultures.

Since the method of TII cell isolation was the most obvious difference between our experiments and those published by other investigators, we hypothesized that the bulk of the measured cytokines seen with LPS treatment of TII cells comes from the contaminating cells within the TII cell preparation. This concern of cell contamination contributing to the overall cytokine response of TII cells has been expressed by other investigators [12]. Co-culture of FACS-isolated TII cells, which have cell purities of 98–99%, with increasing concentrations of alveolar macrophages produced levels of TNF-α that increased in a dose-dependent fashion and were similar to those obtained with stimulated macrophages alone, suggesting that the contaminating macrophages within the non-FACS TII cell preparations account for the majority of the TNF-α expressed when the cells are treated with LPS. IL-6 production, however, was not significantly different in co-cultures of FACS TII cells with macrophages stimulated with LPS, nor did the levels of IL-6 at any FACS TII/macrophage co-culture concentration reach those obtained from studies of LPS-treated non-FACS TII cells (Figure 4A). These findings suggest that there may be cells other than macrophages within the contamination cohort of the non-FACS TII cells that contribute to the LPS-stimulated rise in IL-6 production.

One of the most striking findings in this study was the dramatic increase in TNF-α and IL-6 production in the co-cultures of TI cells and macrophages. Exposing cultured TI cells to conditioned media from LPS-treated macrophages suggested that while TI cells do respond to inflammatory mediators liberated by activated macrophages by increasing TNF-α secretion, cell-cell interaction between macrophages and TI cells is a much more potent factor in modulating TI cell cytokine release. Conditioned media from activated macrophages decreased the levels of TI cell IL-6 compared to LPS-stimulated TI cells alone, while LPS treatment of co-cultures of TI cells and macrophages significantly increased IL-6 production. The IL-6 conditioned media experiments reinforce the notion that cell-cell interaction between TI cells and macrophages is powerful, as co-culture of these cells overcomes the inhibition of IL-6 production in TI cells by inhibitory mediators released by activated macrophages. While the exact cell of origin of the IL-6 in this instance is unclear, these results not only make a strong case for TI cells as key participants in the immune response of the lung, but they also suggest that the interaction between macrophages and TI cells may be equally important.

Studies of pulmonary surfactant in the immune response have suggested that surfactant dampens inflammation, particularly in the face of endotoxin-stimulated lung injury [34], [46], [47], but the mechanism by which this occurs has remained unclear. Surfactant has been shown to decrease cytokine production in whole lungs [48], [49], and this general finding has been attributed to the effects of surfactant on alveolar macrophages and dendritic cells. There have been reports that surfactant may bind members of the TLR-4 pathway or may inhibit the translocation of TLR-4 to lipid raft domains, thus blunting TLR-4-driven cytokine production [33], [50]. Our co-cultures of TII cells and macrophages displayed reduced levels of IL-6 when compared to IL-6 production in LPS-treated macrophages alone, initially suggesting that surfactant may be blunting the IL-6 response. Our findings demonstrated that exogenous surfactant decreased TNF-α and IL-6 production in LPS-treated TI cells, but did not significantly affect cultured TII cells or macrophages. Given that TII cells produce surfactant, additional surfactant may not have conferred any additional biological effects. And while the doses utilized here have been used in other studies, our doses in macrophages may have not been high enough to elicit an inhibitory response [33], [35], [50]. Nevertheless, our studies suggest that there may be a component of cellular interplay between TII cells and macrophages in co-culture that is leading to a reduction in IL-6 production after LPS stimulation. Furthermore, the fact that surfactant decreased cytokine production in LPS-treated TI cells suggests that any portion of the dampened inflammatory cytokine response in the distal lung achieved through the addition of exogenous surfactant may have been due to the actions of surfactant on TI cells alone. Future work in determining how surfactant can affect the immunity of TI cells may lead to a better understanding of how surfactant modulates the immune response of the lung.

The NF-κB pathway is important in stimulating pro-inflammatory cytokines and activating genes that regulate the inflammatory response in multiple different organs, tissue and cells [51]. Up-regulation of NF-κB has been shown to enhance cytokine expression in TII cells but whether a similar mechanism occurs in TI cells was not known [29], [36]–[38]. TI cells exposed to LPS for 10 minutes demonstrated phosphorylated IKKα/β staining that progressively decreased in intensity after 30 minutes, which correlates with the known rapid activation of the NF-κB pathway upon LPS stimulation [38]. In addition, NF-κB transcription was elevated in cultured TI and TII cells stimulated with LPS after 8 hours, further suggesting that this may be one method by which LPS induces cytokine expression in alveolar epithelial cells.

Recently, Yamamoto et al. [52] developed a conditional transgenic mouse model of mutated RelA, an NF-κB protein, throughout the alveolar epithelium and reported that only a small subset of inflammatory mediators was produced by the alveolar epithelium after LPS or *Streptococci pneumoniae* exposure upon comparison of wild type to RelA^Δ/Δ^ mice. While these are very interesting results, comparisons between their study and ours must be made with caution. As is often the case with transgenic mouse models, the relative presence, or absence, of certain targeted genes can vary, even between genotypically similar littermates [53]. Furthermore, species specific differences in the inflammatory response have been well-reported [54]–[58], which may be a primary reason for the discordant results. The authors subjected whole lung samples from their wild type and RelA^Δ/Δ^ mice to PCR array analysis, whereas we focused on isolated rat TI and TII cells. They did study isolated mouse TI cells, but only measured production of CCL20 and CXCL5 mRNA, whereas we measured both transcript and protein expression patterns of various pro-inflammatory cytokines. Nevertheless, despite the differences in study design and methods, the final message culled from both of our reports is largely the same: alveolar TI cells may play an active role in the innate immune response. While the differences in cell-specific cytokine production that we observed are intriguing, there was never the expectation that the alveolar epithelium would produce levels of cytokines that would rival that of immune cells such as alveolar macrophages or dendritic cells. The more relevant question to us was whether or not TI cells could produce cytokines, and thus participate in the innate immune response of the lung.

Our data has shown that TI and TII cells not only respond differently to LPS stimulation, but there exists the possibility that the two alveolar cell types work in concert within their microenvironment to provide a balanced inflammatory response to infection. One could postulate that TI cells may serve to heighten the pro-inflammatory response with enhanced cytokine production, particularly in the presence of alveolar macrophages, while TII cells, through the production of surfactant and relatively modest cytokine expression, provide more of an anti-inflammatory response by trying to contain inflammation. Future investigation into possible mechanisms contributing to the differential inflammatory response of TI and TII cells to LPS, along with more characterization of the alveolar microenvironment during injury, are essential to better understanding the role of the alveolar epithelium and its component cells in the innate immune response of the distal lung. Studies to determine if the responses reported here are stimulus-specific or if the alveolar epithelial cells respond similarly to other respiratory insults such as viruses, gram-positive bacteria or tobacco smoke are also warranted. Given that diseases such as pneumonia and acute respiratory distress syndrome all heavily impact diverse patient populations, elucidating the role of TI and TII cells in the innate immune response could help develop new therapies and treatment options to improve outcomes for all affected patients.
